# What Injured Workers With Complex Claims Look For in Online Communities: Netnographic Analysis

**DOI:** 10.2196/17180

**Published:** 2022-04-07

**Authors:** Michelle Manning Hutson, Sarah M Hosking, Soula Mantalvanos, Michael Berk, Julie Pasco, Trisha Dunning

**Affiliations:** 1 School of Medicine Deakin University Geelong Australia; 2 Pain Train Geelong Australia; 3 Barwon Health Geelong Australia; 4 Department of Medicine – Western Health, Melbourne Medical School The University of Melbourne Melbourne Australia; 5 Department of Epidemiology and Preventive Medicine Monash University Melbourne Australia; 6 Centre for Quality and Patient Safety Research, Barwon Health Partnership School of Nursing and Midwifery Deakin University Geelong Australia

**Keywords:** work-related injury, care coordination, case management, netnography

## Abstract

**Background:**

Improved understanding of social constructs around injury may help insurance case managers to understand how best to support people after injury.

**Objective:**

This study sought to explore what people who sustain work-related injuries may seek from online communities. The study highlights potential opportunities for improved engagement with insurance case management practice.

**Methods:**

An observational netnographic analysis was undertaken on anonymous, publicly available messages posted on Australian message boards. All research data were drawn from anonymous, online communities. A person (author SM) with experience of making a claim through an Australian workers’ compensation system and online engagement was involved in study conception, design, and analysis. Data were analyzed using NVivo12 in an iterative, multistage process including coding, journaling, and member checking. A total of 141 people were engaged in discussion across 47 threads housed on 4 Australian forums.

**Results:**

In this qualitative study, themes emerged from the data, describing how injured workers use online communities to help make decisions, get support, and solve problems. The key motivators for action and engagement were seeking information, connection, or justice. Establishment of relationships was a key mediator of each of these parameters.

**Conclusions:**

Some work-related injuries may involve medical and medicolegal complexity as well as changed lifestyle and routine during convalescence and recovery. The mechanism used by some injured workers to seek information and problem solve suggests a capacity for self-management and self-care after work-related injury. Netnography provides information on a community that may not regularly engage with research because of the complexity of their situation and their vulnerability.

## Introduction

Recovery from work-related injuries involves more than a clear clinical path. Injured workers can also be impacted by psychological and lifestyle factors, which moderate both recovery and return to activities of daily living [1]. Biopsychosocial factors are known to influence recovery outcomes, overlaying other more obvious aspects of recovery such as biomechanics and access to care [2]. Recovery responses are mediated not only by compliance with treatment, but also by the expectations of the treatment team, friends and family, work colleagues, and society at large [3]. Work-related injuries can be costly for organizations, insurers, and individuals, so understanding the experiences of injured workers may help improve care outcomes and reduce costs.

Many individuals making a claim for compensable injuries describe feeling stigmatized [4]. Previous qualitative research studies with injured workers describe some positive experiences, mitigated by a variety of negative experiences [5,6]. Calvey and Jansz [6] highlight opportunities for practice improvement, which largely center around communication and information quality, and maintaining a sense of connection with work teams and treatment teams.

Research with potentially vulnerable injured workers must take into account the impact of power differentials, and digital ethnographic research methods provide a useful way to undertake sensitive observational research addressing experiences and perceptions in a public arena [7]. Qualitative, human-centered web research techniques such as netnography offer a valid way to describe social phenomena in a less invasive, cost-effective manner. Qualitative studies highlight the need to ensure quality communication with workers to promote improved outcomes following work-related injury [8].

This study sought to explore why injured workers participate in online communities, and to understand what injured workers may be seeking to promote or expedite their recovery. This research sought to discern areas for practice improvement for claim management, with a view to improve worker experience and health outcomes. The explorative nature of this work seeks to inform future research directions.

## Methods

### Overview

A person with experience of making a claim through an Australian workers’ compensation system and online engagement (author SM) was involved in study conception, design, and analysis. Material was sourced from a variety of publicly available forums or message boards, all of which had an explicit position statement indicating that information shared on the board or website was publicly available, and all with a policy requiring users to maintain anonymity for themselves and others. Forums were selected as the most appropriate medium on the internet (compared with websites or social media) because of their interactivity and public nature. Forums were identified through a Google search for “work”, “claim”, and the truncated wildcard search stems “injur*” and “comp*”. Forum selection was based upon the search terms; in addition, only forums with a minimum of 5 posts per thread and minimum of 5 threads that discussed work-related injury were selected. Privately held websites that did not have policies on poster anonymity or were not designed as discussion forums were excluded because of the potential risk of reidentification of posters. Anonymous posts from Australian websites were included, based upon either a variant of “Australia” in the forum title or the use of Australia’s URL domain extension (.au). Data were collected from March to June 2019.

Data were analyzed using NVivo12 (QSR International). Code development was an iterative, multistage process, including attribute coding, descriptive coding, and modified versus coding, following a process described by Saldana [9]. In order to become familiar with the data corpus, the complete data corpus was reviewed 4 times before coding began, and a codebook acted as a reference for a priori codes. Analytic memos were used to record research ideas, as a research log and as part of the audit trail. To promote rigor and reliability of results, reflexive journaling was undertaken and member checking occurred with members of the research team and individuals with lived experience of compensable injury, as described by Liamputtong [10]. We used the Standards for Reporting Qualitative Research Checklist when writing our report [11].

### Ethical Considerations

The study design was observational, not participatory, and used only publicly available archival data. The researchers did not elicit information from participants or participate in discussion. We considered the position of Eysenbach and Till [12] in the development of our methodology, aiming to reduce intrusiveness through passive analysis of anonymous, publicly available data. Our intention was to maintain privacy, dignity, and integrity for the posters who participated in the forums, and we excluded forums with fewer than 4 threads addressing the topic of work-related injury to minimize risk of reidentifiability.

## Results

### Overview

Community members who posted in threads were considered “produsers” as they both consumed and generated content in their engagement with the community [13]. Although it is known that active posters, people who visit such forums and do not post (lurkers), and nonusers of online forums may differ significantly, data on the latter two groups was unavailable [14]. The primary sources were 4 individual forums, yielding a total of 47 threads discussing work-related injury. In total, there were 70,206 words in the data corpus. There were 141 individuals interacting within the data corpus, which spanned from 2006-2019. No analysis of changes in the use of social media over time was undertaken. As the study was observational rather than interventional, assessment of change of psychological states over time was not possible. A total of 26 codes (6 a priori, 20 emerging, 19 in vivo) were described, as seen in Table 1.

**Table 1:** Themes emerging from the data corpus.

Coding, themes, and subthemes		
**A priori codes**		
	Advice	
	Claim management	
	Management and treatment	
	Physical to psychological injury	
	Support	
	Venting	
**Emergent codes**		
	**Legal**	
		Entitled
		Legalities
	**Operational**	
		Decision-making
		Financial stressors
		Practical loss
	**Physical, emotional**	
		Experiences of pain
		Grief and loss
		Justice and injustice
		Mindfulness and acceptance
		Suicide risk
		Trust and mistrust
		Validation
	**Relationship development**	
		Humor and black humor
		Mirroring
		Relationships emerging
	**Within and between systems, navigation**	
		Expected responses
		Health literacy
		System design and navigation

### Characteristics of Websites With Injured Worker Communities

The study sought to examine Australian forums; however, the forums were anonymous and thus geographic limitations could not be applied as this would require verification of identity. Forums provided less restrictive character or word limits than other digital spaces. There was an apparent preference for sites where weblog-like dialogue could be shared, rather than performative communities like Instagram, TikTok, or YouTube, as evaluated by number of posts available and amount of interaction achieved by the poster. Private conversations as may be found in private Facebook groups or WhatsApp, Weebo, or Messenger groups were excluded because of their private nature.

Online participants largely communicated with words rather than imagery. Although members of the community often used humor and black humor, they did not appear to use memes or gifs as seen in other online communities. Many within the group used emojis or evocative profile pictures to supplement their text; however, these were not included in the analysis. The lack of use of enriched images such as memes, gifs, or video may indicate the intense desire for anonymity or a potential lack of confidence or comfort with technology within this cohort.

At times, posts were created in threads that appeared to function as a diary, with factual information and little engagement with others; these may provide a historical record, which may be externally validated by providing timestamps or other metadata. An individual worker may be able to identify their own content; however, it would be difficult for employers, investigators, or lawyers to reidentify posters because of the anonymity policy of the boards. This feature allows forums to provide a record or history, acting as a form of documentary evidence for isolated workers. Further to this, Hinton and Hjorth [15] notes some digital content is produced not to elicit or provoke response, but remind the community that an individual is still there, which has particular relevance given the social isolation that can be endemic in this community.

**Figure 1:**
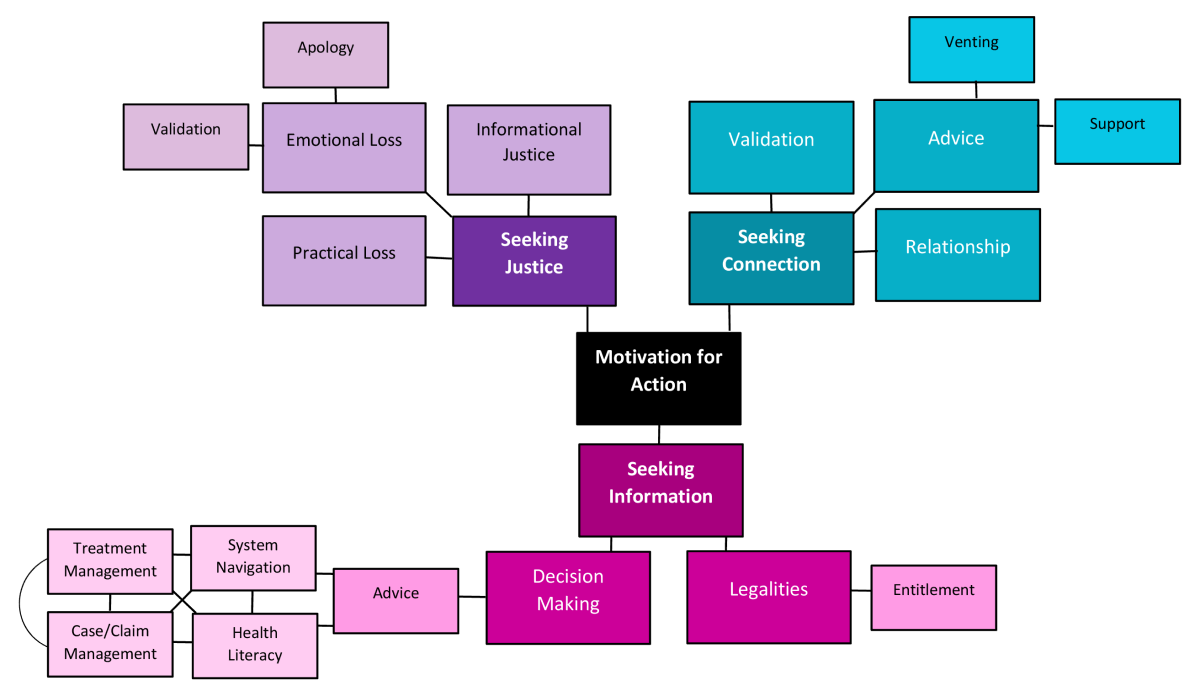
Conceptual framework describing motivation for injured worker community interactions.

A large proportion of individuals described complicated or complex injuries; people with straightforward claims appeared less likely to engage or participate in the communities identified. Those who engaged in these communities were following a model of interaction (Figure 1) that appeared to be underpinned by a need for validation; however, they were consistently seeking justice, validation, or connection, or combinations of justice, validation, and connection.

Others in the community seldom provided advice without also providing validation or empathy to the original poster. This interplay of seeking advice while receiving validation or empathy supported the development and emergence of relationships within groups.

### Seeking Justice

It appeared that individuals desired validation from members of their community—they described acknowledgment of their situation or difficulty by another as validation. Elbers [16] explains that perceptions of fairness may moderate health outcomes for individuals with compensable injuries. Individuals talked generally about the importance of acknowledgment or an apology following a work-related injury (“I’m still hurt and angry because no one will listen to me and try to acknowledge that what I’m going through is real and valid” [B19]).

The individual or organization providing the apology was less important than providing an acknowledgment of the worker’s situation (“I just want to feel validated for my experiences and injury… most injured workers just want an apology… so you can move forward” [Z18]).

Although some sought financial recompense for their injury, many others noted that an acknowledgment or validation of their suffering would go some way toward supporting their recovery. Previous work has noted the importance of empathy in increasing perceptions of interpersonal and informational justice in organizations [17].

### Information

Individuals often noted difficulty with navigating complex medicolegal systems, or alluded to having difficulty with interpreting information provided by insurance companies or the workplace Human Resources department. Some workers displayed frustration directed toward insurers or their agents: “It’s like they [insurance companies] want us to die to avoid paying out” [M17]. Individuals also described experiences of how they are treated as influencing their perceptions of outcomes or mental states: “I feel like dealing with them is going to break me” [K19].

Some posters indicated that they required formalized assistance with navigating complex systems. Although some community members noted problems interacting with lawyers or understanding legal complexities, others recommended engaging a lawyer as an advocate in addition to the treatment and claim management teams:

I have 2 [Psychiatric Independent Medical Examiners], my [Psych] and my doctors reports all saying I’ll never work again. I also have a no win no fee lawyer who is handling everything.

G17

Individuals often described complex circumstances and empathized with others who were trying to navigate the system without support. They often engaged with a discussion of difficulty navigating systems by listening and empathizing rather than by giving advice.

### Connection

Many appeared to be motivated by connection and relationship with other members of the community in an altruistic way (“Sitting with you” [B19]), although others described emotional needs that had been unmet by the system or other stakeholders within the formal system (“the word frustration is inadequate compared to the suffering endured” [O13]). As individuals developed trust and rapport within the community, they turned to other members of the community for justice, information, connection, or a combination of these.

## Discussion

### Principal Findings

In this qualitative study of online communities, the broad themes of justice, information, and connection emerged. Many individuals described how acknowledgement and validation may contribute to or form part of their recovery. For some injured workers, online communities act as a place to find support, to aid decision-making, or to problem solve. The establishment of relationships was a key mediator of each of these parameters.

Oldenburg describes a “third place” outside of home and the workplace, which serves to meet a distinct social and emotional need [18]. Soukup [18] describes online communities as an essential modern “third place,” with online communities acting as a social and emotional surrogate for real-world interaction. Calvey and Jansz [6] describe the consumer-focused service delivery model for provision of care for injured workers, the heart of which is being treated as a person and a partner in activities, and giving and receiving empowerment, trust, and advocacy. The online injured worker community demonstrates features of this model, creating community, seeking and sharing information within their community, and creating a space for occasional levity. The phrase “I feel like” is used often, which has a less declarative tone and invites discourse and new perspectives. Indeed, “I feel like” occurred with twice the frequency within the data corpus as the less emotionally driven “I feel that.” Often individuals prefaced a discussion of intangible concepts by using “I feel like” as a signal, such as “I feel like a forgotten soldier,” when compared with more concrete concepts such as “I feel that my right knee is…” Members of the communities often engaged in relationship building, empathizing, and advising, as evidenced by repetition of terms, mirroring of concepts, and reflection on an aspect of a post before providing further information. This suggests that there is less desire for performative self-expression and more desire for engagement with other members of the group.

In addition to online message boards, other communities exist online. Personal websites such as WorkCover Victim’s Diary or Pudendal Nerve [19] are not anonymous and serve as a diary or record, as well as being a source of information; they are not strictly designed to promote dialogue within the community, although they permit commenting on published articles. In contrast, message boards meet “third place” criteria such as having a sense of community, being accessible, being based on conversation, and including a sense of playfulness.

From a system navigation perspective, there are some health literacy implications: injured workers are actively seeking information and assistance to improve their ability to navigate complex systems. “Straightforward” claims are seldom represented: most describe claims which are medically, operationally, or psychosocially complex (eg, disputed entitlement, unexpected complications, delayed recovery). Many describe having made attempts to have their informational needs met through usual channels such as discussion with stakeholders including their employer, treatment team, or insurer. Further work is required to understand informational needs of injured workers to determine what aspects of formalized information sharing may need to be improved to better facilitate decision-making in an adversarial system—whether this is improved information quality, improved availability, or better timing of information sharing.

**Figure 2:**
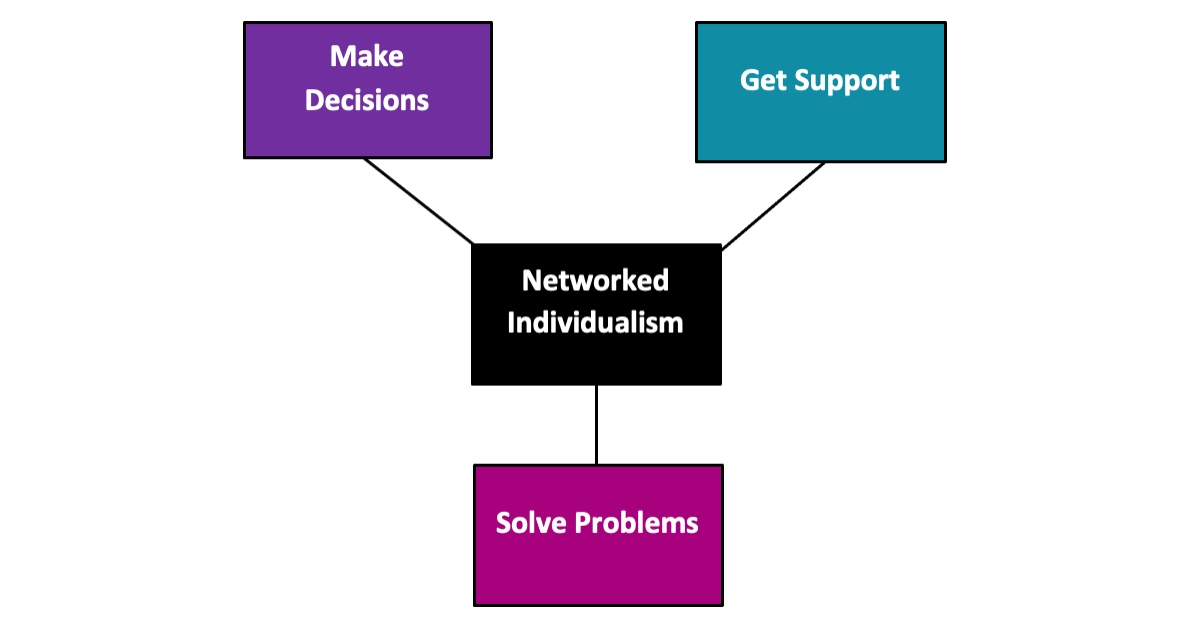
Networked individualism in practice (adapted from Wellman [20]).

Hinton [15] described Wellman’s [20] “networked individualism” (Figure 2) as a process through which individuals participate in the online world to make decisions, solve problems, or get support. This pattern of participation and engagement is apparent within the injured worker “produser” community, where individuals are seeking something beyond even “person-centered care” or “circles of support”—they are meaningfully engaged with a broader community of virtual strangers who provide information, collaboration, and validation. “Produsers” who act as networked individuals may be demonstrating their capacity for self-actualization and self-management of their recovery. Previous case management research has shown that in a consumer-directed model of care, the locus of power shifts from the case manager toward the individual [21]. As individuals move toward self-management, case managers provide information, enable self-empowerment, build capacity, and provide support to individuals to self-manage their care needs [21]. This research elicits the possibility that some injured workers may benefit from a style of case management that centers the locus of control away from the organization and supports proactive decision-making by injured workers.

Although netnographic analysis permits a rapid and broad analysis of the online milieu, there are limitations inherent in using an observational technique. We did not actively engage participants in research; rather, we observed what was currently available online. Accordingly, not all workers were represented and it is not possible to determine whether there were underrepresented groups within the cohort. This study only investigates posters, not lurkers or nonusers, and the findings cannot be generalized. In addition, due to the specific nature of the Australian system, the results cannot necessarily be generalized to other systems and countries. Therefore, the findings must be interpreted and applied with great caution. This research has a role in informing practice improvement opportunities for case managers but should not be taken into account in actuarial models without further analysis.

### Conclusions

An observational netnography of injured workers revealed how a sample of injured workers with complex care needs use networked individualism online to make decisions, get support, and solve problems. The study revealed broad themes of seeking justice, information, and connection, all of which were underpinned by a desire to find validation. This mechanism for information seeking and problem solving demonstrates a meaningful engagement with community and a capacity for self-management of their own care, as distinct from traditional models of insurance case management. The actuarial implications of these findings have not been explored, and accordingly the findings should be interpreted with cautious optimism in any scheme translation.

## Data Availability

The data that support the findings of this study are available from the corresponding author, MMH (ORCID: 0000-0002-3312-462X), upon reasonable request.
